# 20(S)-Ginsenoside Rg_3_ is a novel inhibitor of autophagy and sensitizes hepatocellular carcinoma to doxorubicin

**DOI:** 10.18632/oncotarget.2034

**Published:** 2014-05-28

**Authors:** Dong-Gun Kim, Kyung Hee Jung, Da-Gyum Lee, Jung-Ho Yoon, Kyeong Sook Choi, Sung Won Kwon, Han-Ming Shen, Michael J. Morgan, Soon-Sun Hong, You-Sun Kim

**Affiliations:** ^1^ Department of Biochemistry and Department of Biomedical Sciences, Ajou University School of Medicine, Suwon; ^2^ College of Medicine, Inha University, Incheon; ^3^ College of Pharmacy and Research Institute of Pharmaceutical Sciences, Seoul National University, Seoul, Korea; ^4^ Saw Swee Hock School of Public Health, National University of Singapore, Singapore; ^5^ Department of Pharmacology, University of Colorado School of Medicine, Aurora, Colorado

**Keywords:** Rg_3_, autophagy, HCC, doxorubicin, cell death

## Abstract

Hepatocellular carcinoma (HCC) is the second leading cause of cancer-related deaths worldwide. High mortality from HCC is mainly due to widespread prevalence and the lack of effective treatment, since systemic chemotherapy is ineffective, while the targeted agent Sorafenib extends median survival only briefly. The steroidal saponin 20(S)-ginsenoside Rg_3_ from *Panax ginseng* C.A. Meyer is proposed to chemosensitize to various therapeutic drugs through an unknown mechanism. Since autophagy often serves as cell survival mechanism in cancer cells exposed to chemotherapeutic agents, we examined the ability of Rg_3_ to inhibit autophagy and chemosensitize HCC cell lines to doxorubicin *in vitro.* We show that Rg_3_ inhibits late stage autophagy, possibly through changes in gene expression. Doxorubicin-induced autophagy plays a protective role in HCC cells, and therefore Rg_3_ treatment synergizes with doxorubicin to kill HCC cell lines, but the combination is relatively nontoxic in normal liver cells. In addition, Rg_3_ was well-tolerated in mice and synergized with doxorubicin to inhibit tumor growth in HCC xenografts in vivo. Since novel *in vivo* inhibitors of autophagy are desirable for clinical use, we propose that Rg3 is such a compound, and that combination therapy with classical chemotherapeutic drugs may represent an effective therapeutic strategy for HCC treatment.

## INTRODUCTION

Autophagy is a catabolic cellular degradation response to various stresses whereby cellular proteins, organelles and cytoplasm are engulfed, digested and recycled to sustain cellular metabolism [1-3]. Autophagy has diverse biological functions, and plays a role in many physiological and pathological processes, including cancer, neurodegenerative diseases, and immunity [4]. In cancer, it plays important functions both in cell death and survival, as well as tumor progression [5-7]. Autophagy is generally recognized as a pro-survival mechanism in response to a number of stressors, including cancer chemotherapeutics, and therefore, inhibition of autophagy sensitizes to cell death in many cases. Conversely, under certain conditions, the overactivation of autophagy by some stimuli promotes cell death [8]. In most situations, however, even when there is real increase of autophagic flux in the dying cells, enhanced autophagy may indicate an attempt by the cell to prevent itself from dying [9-11]. Careful study is therefore needed to determine the function of autophagy in cell death and survival under different conditions.

Hepatocellular carcinoma (HCC) is one of the most common solid tumors and the second leading cause of cancer-related deaths worldwide. The high mortality rate in HCC patients is mainly due to the lack of effective treatment, especially for those with advanced disease. To date, systemic therapy with classical cytotoxic chemotherapy is generally poorly tolerated and ineffective for HCC, and effective therapy with targeted agents is currently limited to the multi-kinase inhibitor Sorafenib. For this reason, new and more effective therapeutic options for this form of cancer were eagerly sought after. Combination therapies are being developed as a more promising therapeutic strategy in HCC treatment [12, 13]. Since autophagy is often a pro-survival response to chemotherapeutic drugs, combination therapies where autophagy is inhibited during chemotherapy may be a good therapeutic strategy. Inhibition of autophagy may especially be useful in sensitizing HCC cells to both sorafenib and other classical chemotherapeutic treatment [14, 15]. Currently, chloroquine, and its derivative, hydroxychloroquine, are the only FDA approved drugs that are currently used in patients as inhibitors of autophagy. Additional clinical agents that prevent autophagy are currently being explored, but none have yet been approved for use in the clinic.

Ginsenoside Rg_3_ extracted from the steamed *Panax ginseng* C.A. Meyer is one of a diverse group of steroidal saponins with high pharmacological activity [16]. Stereospecific effects have been observed from this compound, with the 20(R) enantiomer, for instance, being more active as an antioxidant and in its promotion of the immune response [17, 18], and the 20(S) having a greater potential anti-diabetic activity [19]. While the 20(R) enantiomer of this compound is poorly soluble, the 20(S) enantiomer has a much higher solubility, thus making it more suitable for pharmaceutical development. The 20(S) Rg_3_ stereoisomer has also been shown to be remarkably non-toxic and well-tolerated in mice, rats, and dogs [20-22]. Several findings suggest that Rg_3_ may increase the efficacy of cancer chemotherapy [16, 22-24]. A number of molecular mechanisms have been proposed for such anti-cancer function, among which are inhibitory effects on NF-κB and AP-1 activity [25], and the down-regulation of angiogenesis associated with VEGF expression [23, 24, 26-28]. However, the related ginseng-derived ginsenosides Rb1 and Rk1 have been shown to have effects on autophagy. Rb1, for instance, has an influence on the activation of autophagy in glutamate-injured neurons [29], while the anti-tumor activity of Rk1 is thought to be due to its ability to modulate autophagy [30]. The mechanism of autophagy modulation by ginsenosides is unclear and there is currently no published data showing that 20(S)-ginsenoside Rg_3_ affects autophagy.

In the present study, we investigated the effect of the 20(S)-ginsenoside Rg_3_ on modulation of autophagy in hepatocellular carcinoma cell lines to evaluate whether this is relevant to the sensitization effect of Rg_3_ on cell death induced by doxorubicin, a commonly used cancer therapeutic agent in a wide range of cancers. Our data show that Rg_3_ is capable of inhibiting autophagic flux via suppressing late stage autophagosome maturation or degradation. Because doxorubicin- induced autophagy has a pro-survival function, Rg_3_-induced suppression of autophagy contributes to its sensitization of HCC cells to doxorubicin-induced cell death. Synergism of Rg_3_ and doxorubicin was confirmed *in vivo* using a mouse xenograft model. Our data therefore suggests that the inhibitory effect of Rg_3_ on autophagy is functionally related to its sensitization effect on doxorubicin-induced cell death and that such a combination therapy could serve as a novel strategy in treating HCC.

## RESULTS

### Rg_3_ enhances LC3 II stability and puncta formation in a dose- and time-dependent manner in hepatocellular carcinoma cells

**Figure 1:**
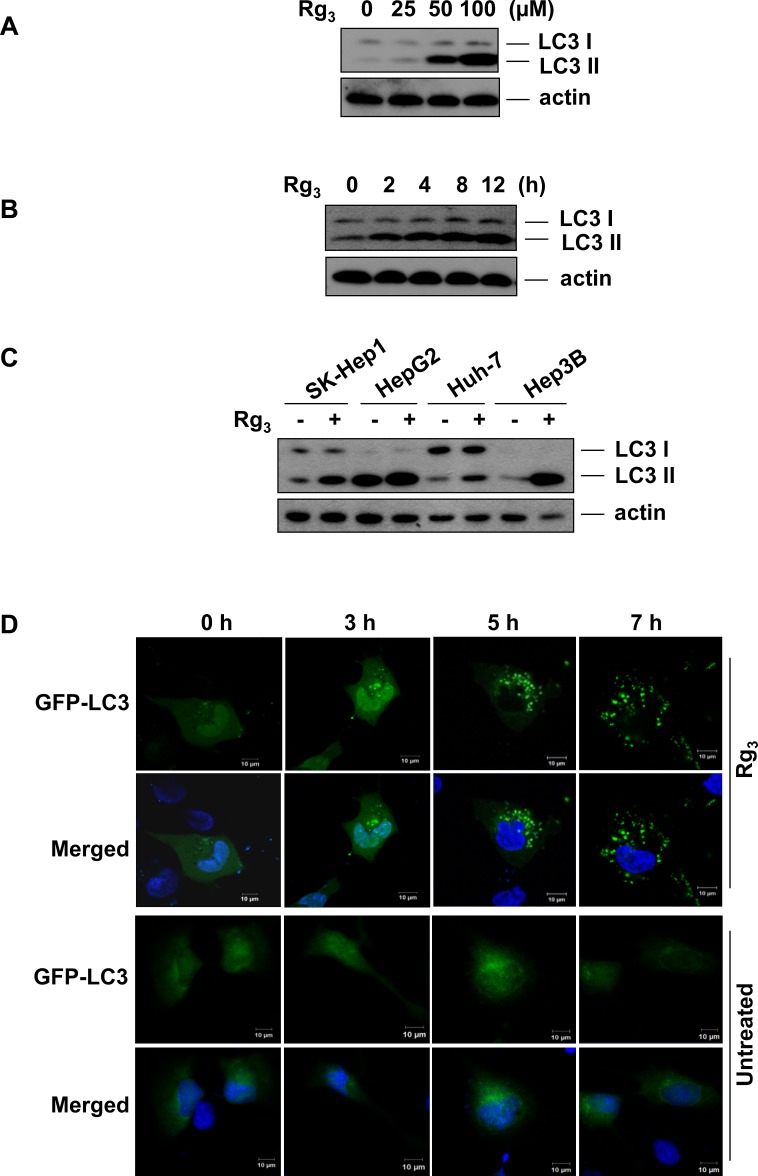
Rg_3_ induces LC3 II accumulation in a dose- and time- dependent manner **A & B.** Rg_3_ promotes LC3 II accumulation. SK-Hep1 cells were treated for 12 h with different concentrations of Rg_3_ and cell lysates were analyzed by western blot using anti-LC3 antibody (A), or cells were treated for indicated time points with 100 μM of Rg_3_ and cell lysates were analyzed by western blot (B). **C.** Rg_3_ induces LC3 II accumulation in multiple hepatocellular carcinoma cell lines. Four different hepatocellular carcinoma cells were treated with 100 μM Rg_3_ for 12 h and cells lysates were analyzed by western blot. **D.** Rg_3_ treatment markedly increases GFP-LC3 punctuation as observed by confocal microscopy. Confocal images show SK-Hep1 cells with transient expression of GFP-LC3 treated with 100 μM Rg_3_ (or left untreated) for the indicated time points and stained with DAPI.

Several earlier studies have suggested that Rg_3_ may have anti-tumor activity but its mechanism of action is unclear. We examined the effect of Rg_3_ on autophagy by measuring LC3 II protein levels in Rg_3_-treated cells. Rg_3_ treatment of SK-Hep1 hepatocellular carcinoma cells increased the amount of LC3 II protein in a dose- and time-dependent manner (Figure 1A and 1B). To determine whether this effect was specific, we compared the effect of Rg_3_ on 4 different hepatocellular carcinoma cell lines (Figure 1C) and two different lung cancer cell lines (Figure S1). All cell lines tested show similar trends in LC3 II protein levels following Rg_3_ treatment, suggesting that the effect of Rg_3_ on autophagy is not cell type specific. Rg_3_ treatment also markedly increased GFP-LC3 puncta formation in SK-Hep1 cells (Figure 1D) and in HepG2 cells (Figure S2), confirming that Rg_3_ treatment affects autophagy.

We verified that the Rg_3_-induced increase in LC3 II formation and GFP-LC3 puncta are due to Rg_3_ effects on the ATG5-dependent autophagic pathway by using Tet-off Atg5−/− MEF cells in which Atg5 and GFP-LC3 are stably expressed [31]. In this system, Atg5 gene expression is abolished following treatment with doxycycline. Rg_3_ enhanced LC3 II formation in MEF cells in the presence of Atg5, but not in its absence (Figure S3A). Consistent with the LC3 II conversion data, the punctuation/aggregation of GFP-LC3 was only induced in cells expressing Atg5, but not in cells without Atg5 (Figure S3B).

### Rg_3_ inhibits autophagic flux in the late stages of autophagy

**Figure 2:**
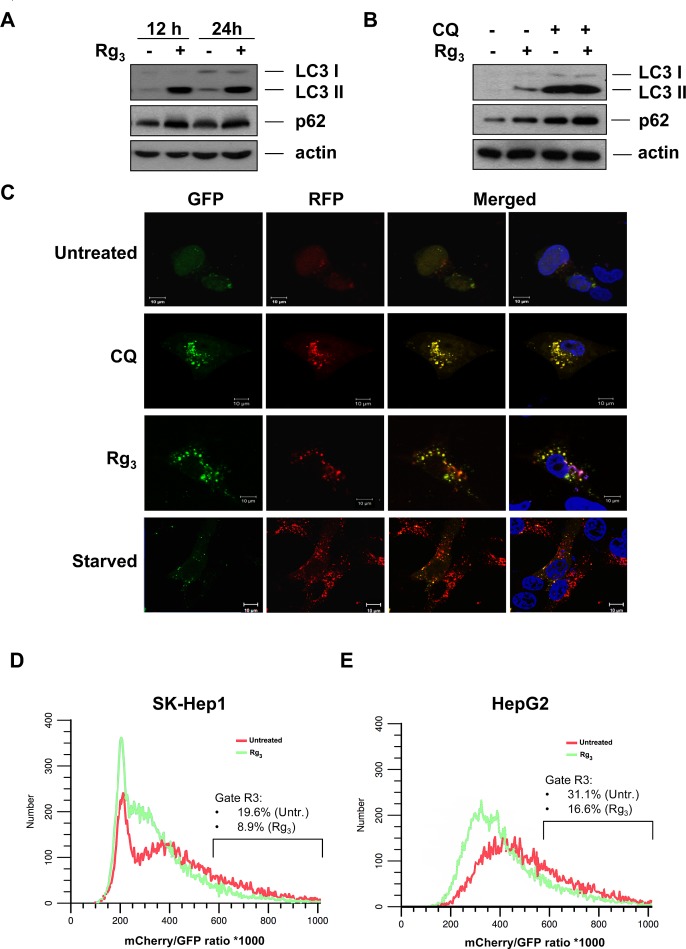
Rg_3_ prevents autophagic flux A. Rg_3_ promotes accumulation of LC3II and autophagic cargo protein p62/SQSTM1. SK-Hep1 cells were treated with 100 μM Rg_3_ for the indicated time periods and cell lysates were collected and subjected to western blot. **B.** Rg_3_ inhibits autophagic flux. SK-Hep1 cells were pretreated with chloroquine (CQ, 10 μM) for 30 min, followed by treatment with 100 μM Rg_3_ for another 12 h and cell lysates were collected and subjected to western blot. **C.,D.,E.** Rg_3_ inhibits completion of autophagy. C. SK-Hep1 cells were transiently transfected with GFP-mRFP-LC3 and cells were treated with 100 μM Rg_3_ for 7 h or CQ (10 μM) for 7 h, or HBSS for 8 h. At the end of treatment, cells were observed for the change of both green and red fluorescence using a confocal microscope. **D.** SK-Hep1 cells were stably infected with GFP-mRFP-LC3 lentivirus and treated overnight with 100 μM Rg_3_. The Red/Green fluorescence ratio was monitored by flow cytometry. E. HepG2 cells were stably infected with GFP-mRFP-LC3 lentivirus and treated overnight with 100 μM Rg_3_. The Red/Green fluorescence ratio was monitored by flow cytometry.

An increase in LC3 II in response to a drug may represent either increased generation of autophagosomes and/or a blockage in autophagosomal maturation and degradation [32-35]. The multifunctional cargo protein, p62/SQSTM1, binds to LC3 and is degraded with its cargo within autolysosomes [36]. Thus, a reduction of p62 can be regarded as a marker for an increase in autophagic flux [34, 37]. However, Rg_3_ treatment rather led to an increase in p62 (Figure 2A, S4). Stabilization of p62 levels was accompanied by additional ubiquitination, suggesting that Rg_3_ inhibits its degradation without interfering with the normal ubiquitination process (Figure S4A-B). Additionally, when cells were treated with chloroquine (CQ), which blocks late stage autophagy by impairing lysosomal acidification, Rg_3_ did not substantially enhance the LC3 II and p62 protein levels (Figure 2B). This suggests that the increased LC3 II conversion and GFP-LC3 puncta formation in Rg_3_-treated cells is not due to enhanced autophagy, but is rather due to suppression of the late maturation or degradation stage of autophagy, resulting in a block in autophagic flux.

In order to further test whether the effect of Rg_3_ is due to suppressed autophagic flux, we utilized a tandem labeled GFP-mRFP-LC3 construct (tfLC3). The tfLC3 construct has been reported to be a useful tool for examining autophagosome maturation and autolysosome formation, and is particularly helpful in distinguishing increased autophagy from a block in autophagic flux [38, 39]. In this assay, since mRFP is much more resistant to quenching than GFP in the acidic environment of the lysosome, if the autolysosome maturation proceeds normally or flux is increased, this is indicated by additional or substantially increased red-only puncta. In contrast, if the autophagosome does not fuse with lysosome or lysosomal function is blocked, most of the puncta should exhibit both red and green fluorescence and thus appear to be yellow in merged images [38]. Rg_3_ treatment of SK-Hep1 cells transiently transfected with tfLC3 led to formation both GFP and mRFP punctuation that extensively co-localize with each other and thus appear yellow, suggesting that upon Rg_3_ treatment the autophagosomes do not fuse with lysosomes or that lysosomal function is impaired (Figure 2C). In contrast, induction of autophagy by nutrient starvation led to the production of large amounts of red-only puncta, while the Rg_3_ effect was similar to CQ treatment, which blocks lysosomal function, indicating that Rg_3_ prevents autophagic flux, and does not increase it (Figure 2C). In both SK-Hep1 and HepG2 cells stably expressing tfLC, we observed a decreased mCherry/GFP fluorescence ratio in Rg_3_-treated cells as measured by flow cytometry, indicating that Rg_3_ reduced the amount of autophagic flux in both cell lines (Figure 2D-E). Rg_3_ treatment prevented the starvation-induced degradation of LC3II in both SK-Hep1 and HepG2 cells (Figure S5A). Additional confirmation that Rg_3_ was capable to some extent of inhibiting a strong autophagic stimulus was shown by a decreased mCherry/GFP fluorescence ratio in Rg_3_ pretreated cells that were then additionally treated with EBSS (Figure S5B), although long term co-treatment was somewhat toxic. Together with the previous data, this suggests Rg_3_ suppresses autophagic flux by interfering with late-stage autophagy.

### Doxorubicin induces autophagy in hepatocellular carcinoma cells

**Figure 3:**
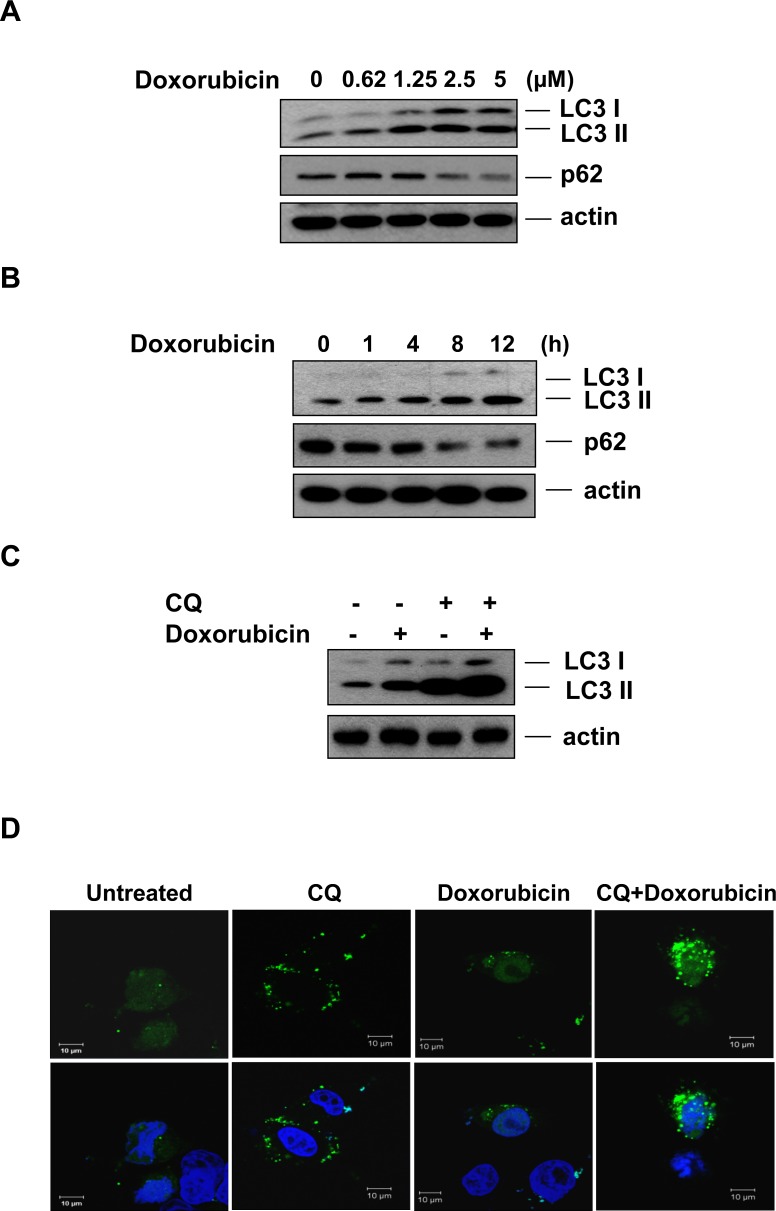
Doxorubicin induces autophagy in hepatocellular carcinoma cells **A & B.** Doxorubicin induces autophagy in hepatocellular carcinoma cells. SK-Hep1 cells were treated with doxorubicin for the indicated concentrations for 12 h (A) or the indicated time periods at a concentration of 2.5 μM (B) and cell lysates were subjected to western blotting. **C.** LC3 turnover assay in doxorubicin induced autophagy. SK-Hep1 cells were pretreated with CQ (10 μM) for 30 min, followed by doxorubicin (2.5 μM) for another 12 h, and cell lysates were collected and subject to western blot. **D.** Doxorubicin induces GFP-LC3 puncta formation in hepatocellular carcinoma cells. SK-Hep1 cells were treated with doxorubicin (2.5 μM) or CQ (10 μM) plus doxorubicin for 7 h and cells were examined with a confocal microscope for GFP-LC3 punctuation/aggregation.

Several studies have suggested that modulation of autophagy can sensitize cancer cells to DNA damage agents [40, 41]. Since our data suggest that Rg_3_ treatment can suppress autophagic flux, we investigated whether Rg_3_ treatment could sensitize to cancer cell death induced by DNA damage. Doxorubicin is a commonly used anticancer drug that causes DNA damage and kills cancer cells mainly by apoptosis [42]. However, doxorubicin-induced cardiotoxicity is also associated with the depletion of a variety of long- and short-lived proteins [43-48], indicating an activation of protein degradation systems including the ubiquitin proteasome system [49, 50]. As shown in Figure 3A and 3B, doxorubicin induced LC3 II production and a decrease in p62 levels in a dose- and time-dependent manner in SK-Hep1 cells. Moreover, CQ further enhanced the amount of LC3 II in cells treated with doxorubicin (Figure 3C), indicating that doxorubicin caused increased autophagic flux. Similarly, doxorubicin induced GFP-LC3 puncta formation (Figure 3D), further indicating that doxorubicin is capable of inducing autophagy in hepatocellular carcinoma cells.

### Autophagy induced by doxorubicin serves as a pro-survival mechanism

**Figure 4:**
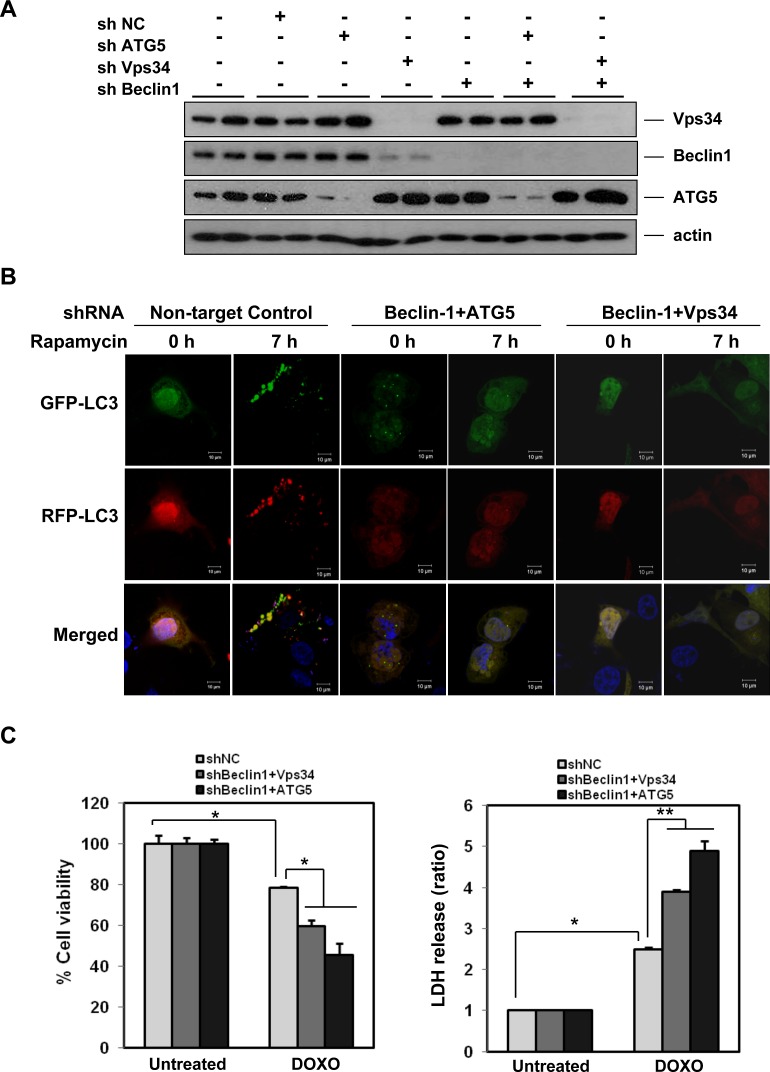
Autophagy has a pro-survival role in doxorubicin-induced cell death **A.** Knock-down of autophagy-related genes. SK-Hep1 cells were infected with a combination of lentivirus encoding Beclin-1, ATG5, or Vps34 shRNAs, or combinations thereof. After selection with puromycin (2 μg/mL), lysates were examined for knockdown by western blotting. **B.** Effects of knock-down of Beclin-1/Atg5 or Beclin-1/Vps34 in doxorubicin-induced autophagy. Inhibition of autophagy in cells from (A) was confirmed by examination of transiently expressed GFP-mRFP-LC3 in the presence of autophagy induction by rapamycin (0.5 μM) for 7 h. **C.** Suppression of autophagy by knock-down of Beclin-1/Atg5 or Beclin-1/Vps34 significantly enhances doxorubicin-induced cell death. Cells from (A) were treated with doxorubicin (2.5 μM) for 18 h and cell viability was analyzed by MTT assay (left panel) and LDH release (right panel). Results are averages +/− SEM. (*p<0.001, **p<0.005)

Next we sought to determine whether the doxorubicin-induced autophagy serves as a pro-survival or pro-death function in HCC cells, since some reports have suggested that positive modulation of autophagy is associated with increased doxorubicin toxicity [51]. We therefore made stable shRNA cell lines in SK-Hep1 (Figure 4) and HepG2 (Figure S6) cells with the double knock-down of Beclin-1 and ATG5 or Beclin-1 and Vps34. Knock-down was confirmed by western blotting in the SK-Hep1 (Figure 4A) and HepG2 (Figure S6A) cells. Rapamycin-induced autophagy was inhibited in double knock-down cells as evidenced by the lack of Green or Red puncta formation in the tandem labeled LC3-transfected knockdown cells (Figure 4B). Suppression of autophagy by Beclin-1/ATG5 or Beclin-1/Vps34 knock-down in SK-Hep1 cells augmented doxorubicin-induced cell death as measured by both MTT and LDH release assays, indicating a pro-survival role for autophagy in doxorubicin-induced cell death (Figure 4C). Similarly, the HepG2 cells with knockdown were sensitized to doxorubicin-induced cell death (Figure S6B). Additionally, inhibition of autophagy with CQ potentiated doxorubicin-induced cell death in HepG2 cells (data not shown), further suggesting that autophagy may have a protective effect against doxorubicin toxicity.

### Rg_3_ sensitizes doxorubicin-induced cell death via suppression of autophagic flux

**Figure 5:**
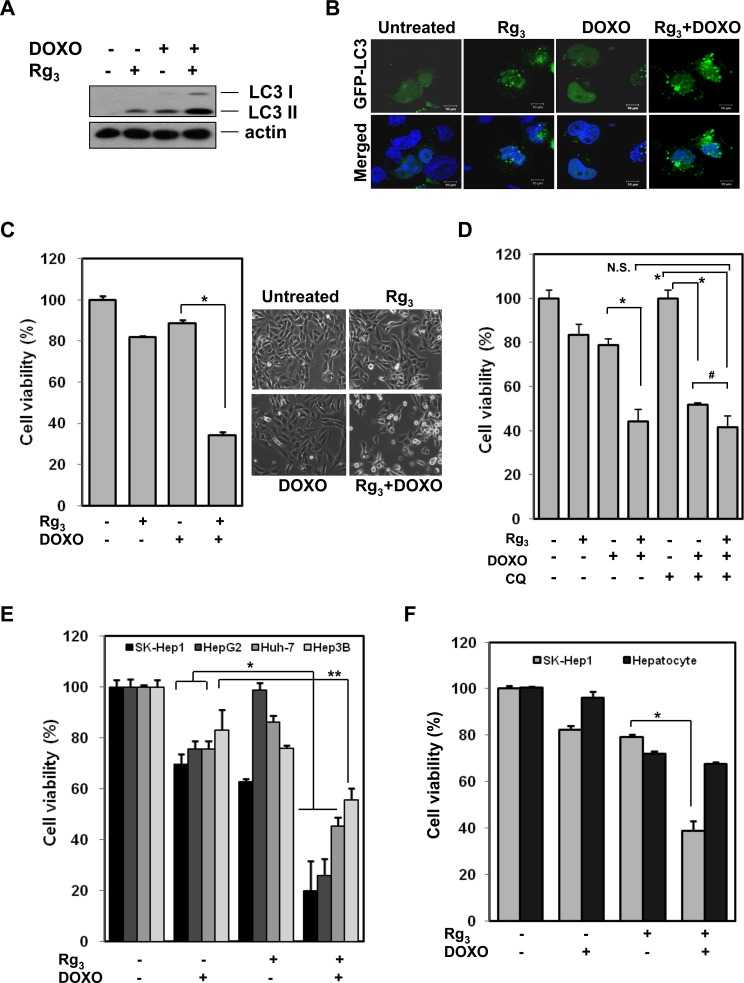
Rg_3_ sensitizes doxorubicin-induced cell death via suppression of autophagy **A.** LC3 turnover assay in Rg_3_ induced autophagy. SK-Hep1 cells were pretreated with Rg_3_ for 30 min, followed by doxorubicin (2.5 μM) for another 12 h, cell lysates were collected and subjected to western blot. **B.** The combination of Rg_3_ and doxorubicin markedly increases GFP-LC3 punctuation. Cells were transfected with GFP-LC3 and treated with Rg_3_ (100 μM), doxorubicin (2.5 μM), or Rg_3_ + doxorubicin for 7 h. Cells were examined with a confocal microscope for GFP-LC3 punctuation/aggregation. **C.** Rg_3_ sensitizes to DNA-damage-induced cell death. SK-Hep1 cells were pretreated with Rg_3_ for 30 min, followed by doxorubicin for another 18 h and cells viability was an analyzed by MTT assay (Left panel) and representative images were taken by a phase-contrast microscope (Right panel). Results are averages +/− SEM. (*p<0.005) **D.** Cell viability (MTT) of SK-Hep1 cells pretreated with CQ (10 μM) or/and Rg_3_ (100 μM) for 30 min, followed by doxorubicin (2.5 μM) for 18 h. Results are averages +/− SEM. (*p<0.001, ^#^p<0.05) **E & F.** Rg_3_ plus doxorubicin-induced cell death is specific to cancer cells. **E.** Four different hepatocellular carcinoma cells were treated with combination of Rg_3_ (100 μM) and doxorubicin (2.5 μM) for 18 h and cells viability was an analyzed by MTT assay. Results are averages +/− SEM. (*p<0.001, **p<0.005). **F.** Human normal liver cells, HL 7702 cells and SK-Hep1 cells were treated with Rg_3_ (100 μM), doxorubicin (2.5 μM), or Rg_3_ plus doxorubicin for 18 h. Cell viability was an analyzed by MTT assay. Results are averages +/− SEM. (p<0.005)

Our data suggest that autophagy serves as a pro-survival function in doxorubicin-induced cell death, whereas Rg3 suppresses autophagic flux. We therefore tested whether the suppression of autophagic flux by Rg_3_ would sensitize hepatocellular carcinoma cells to doxorubicin-induced cell death. Rg_3_ enhanced doxorubicin-induced LC3 II levels (Figure 5A) and GFP-LC3 puncta (Figure 5B) in a manner similar to chloroquine (see previous figures), again suggesting that Rg_3_ blocks doxorubicin-induced autophagic flux at a late stage. When SK-Hep1 cells were pretreated with Rg_3_ for 30 min followed by a low cytotoxic concentration of doxorubicin for 18 h, cells underwent dramatic sensitization to doxorubicin-induced cell death (Figure 5C). Similar results were obtained in HepG2 cells (Figure S7). While both CQ and Rg_3_ could each sensitize cells to doxorubicin on their own, the addition of CQ together with Rg_3_ had no additional sensitizing effect on doxorubicin-induced toxicity compared to Rg_3_ alone (Figure 5D, Figure S7). This indicates that CQ and Rg_3_ sensitize cells to doxorubicin largely though the same mechanism, although addition of Rg_3_ gave some slight additional doxorubicin sensitization compared to CQ alone (Figure 5D, Figure S7). Collectively, these results indicate that autophagy plays a pro-survival role in doxorubicin-induced cell death and that Rg_3_ is able to sensitize HCC cells to doxorubicin-induced cell death in large part through the suppression of autophagy. This sensitization was observed in all the hepatocellular carcinoma cell lines tested (Figure 5E), but when the normal human liver cell line, HL 7702 was treated with Doxorubicin and Rg_3_, no sensitization was seen (Figure 5F), suggesting that Rg_3_ synergism with doxorubicin is selective to cancer cells.

### Rg_3_ combined with doxorubicin promotes caspase-independent cell death

**Figure 6:**
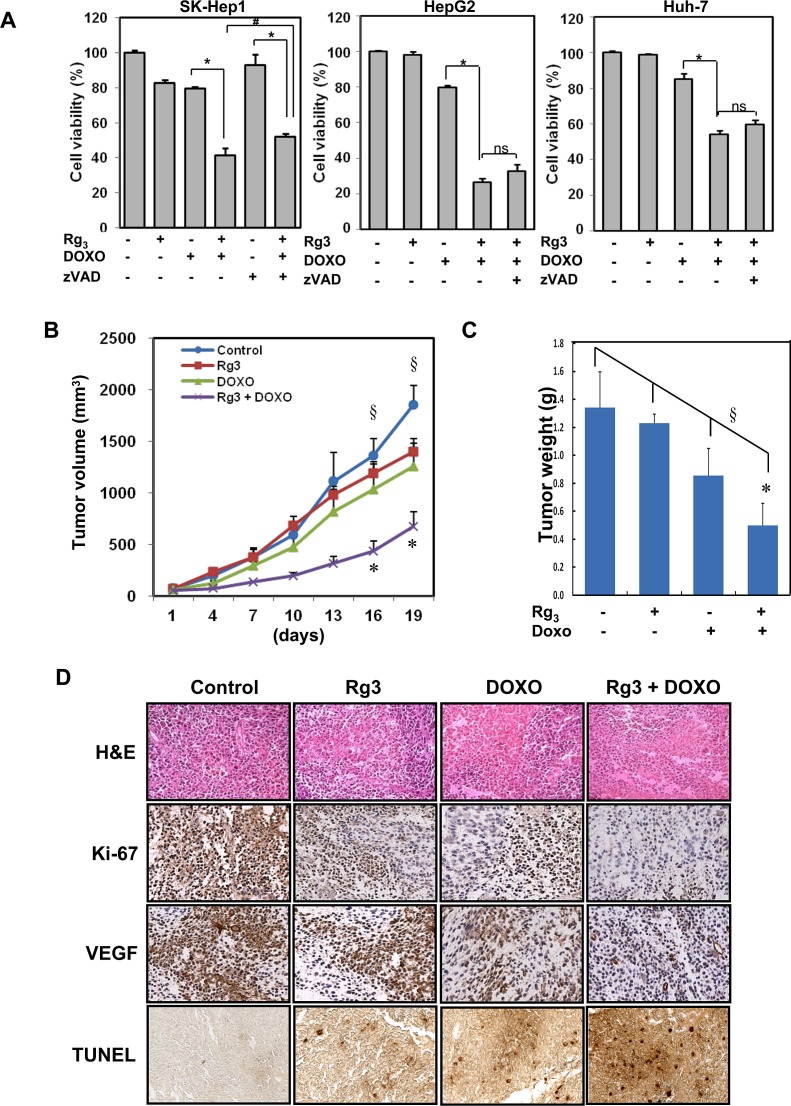
The combination of Rg_3_ plus doxorubicin inhibits hepatoma growth in a mouse xenograft model A. SK-Hep1, HepG2, or Huh-7 cells were treated with Rg_3_ (100 μM), doxorubicin (2.5 μM), or the combination Rg_3_ + doxorubicin with or without zVAD (20 μM) for 18 h and cell viability was an analyzed by MTT assay. Results are averages +/− SEM. (*p<0.001, ^#^p<0.05, ns, non-significant) **B & C.** Effect of Rg_3_ plus doxorubicin on Huh-7 hepatocellular carcinoma cell xenograft in athymic BALB/c nude mice. Huh-7 tumors were established subcutaneously and treated with Rg_3_, doxorubicin or Rg_3_ plus doxorubicin for 19 days. **B.** Tumor volume was monitored. ^§^ANOVA indicates a significance of p= 0.05. *T-Test indicates doxorubicin/Rg_3_ tumor volume is significantly different compared with control (p<0.05), while Rg3 or doxorubicin alone groups are not (p>0.3). **C.** At the end of treatment mice were killed and tumor weight was measured. ^§^ANOVA indicates a significance of p= 0.02 for the experiment. *T-Test indicates combination Doxorubicin/Rg_3_ tumor volume is significantly different compared with control (p<0.05), while Rg3 or doxorubicin alone groups are not statistically significant (p>0.15). **D.** H & E staining for tumor section of mice receiving the indicated treatment. Sections were also stained for Ki-67 and VEGF. Bottom panel shows detection of cell death in the sections by TUNEL assay.

We examined the nature of cell death induced by the combination of Rg_3_ and doxorubicin in HCC cells by pretreating the cells with Rg_3_ for 30 min followed by doxorubicin treatment for designated durations. A minimal amount of caspase-3 cleavage was seen in some of the dying cells treated with Rg_3_ and doxorubicin when compared to the TRAIL induced caspase-3 cleavage (Figure S8A). However, some PARP cleavage could be detected in HepG2 and Huh-7, but in not SK-Hep1 cells, suggesting some caspase activity is present (Figure S8A). However, consistent with limited evidence for caspase activation, when the three cell types were treated with a general caspase inhibitor (zVAD-FMK), zVAD had only a slight effect on cell death induced by the combination of Rg_3_ and doxorubicin (Figure 6A), indicating that the cells were primarily dying a caspase-independent cell death. Since we observed little caspase activation, and since Rg_3_ has been previously reported to induce caspase activation in the colon cancer cell line HT-29 [52], we tested the Rg_3_ and doxorubicin combination in this cell line to see if caspase activation would be more prevalent in this cell type. We were unable to detect either PARP or caspase-3 cleavage in these cells, and zVAD likewise failed to protect the cells from cell death (Figure S8B-C). Thus, Rg_3_ and doxorubicin- induced cell death seems to be a largely a caspase-independent process. Interestingly, the RIPK1 inhibitor necrostatin-1, which is inhibitor of programmed necrotic cell death [53, 54], was also unable to block cell death by combination of Rg_3_ and doxorubicin (Figure S8D), thus suggesting that necroptosis also did not substantially contribute to cell death in this system. Consistent with this lack of effect of necrostatin, all of the HCC cell lines that we used lacked expression of RIP3 (Figure S8E), an essential component of the necroptotic machinery.

To determine whether changes in gene expression were required for the ability of Rg_3_ to inhibit autophagy, we pre-treated cells with cycloheximide (CHX) to inhibit protein synthesis, before inhibiting autophagy. A slight amount of autophagy was induced by CHX addition. While bafilomycin A1 was capable of inhibiting autophagy in CHX-treated cells as shown by a decreased mCherry/GFP fluorescence ratio in Rg_3_ pretreated cells (Figure S9A), when cells were treated with CHX, Rg_3_ could no longer shift the mCherry/GFP fluorescence ratio (Figure S9B). This suggests that gene expression changes are necessary for Rg_3_ to inhibit autophagy. We have previously shown that Rg_3_ sensitizes to TRAIL-induced apoptosis by inducing the expression of the transcription factor CHOP, which then upregulates the transcription of the DR5 receptor for TRAIL [22]. However, CHOP has also been shown to modulate autophagy, and in some cases appears to act as a repressor autophagy [55, 56]. To test whether induction of CHOP is required for Rg_3_ inhibition of autophagy, we knocked down CHOP in HepG2 cells and treated the cells with Rg_3_. Knock-down of CHOP led to increased basal levels of LC3II (Figure S9C). In the absence of CHOP, Rg_3_ treatment did not result in any addition increase of LCII, suggesting that Rg_3_ may inhibit autophagy at least in part through CHOP upregulation (Figure S9C). Knockdown of CHOP repressed the death of HepG2 cells by the Rg_3_-doxorubicin combination at early timepoints (Figure S9C), suggesting upregulation of CHOP is necessary for Rg_3_ sensitization to doxorubicin. However, unlike the potentiation of TRAIL signaling by Rg_3_, the upregulation of DR5 was not necessary for doxorubicin sensitization, and DR5 knockdown actually increased doxorubicin cytotoxicity (Figure S9E).

### Rg_3_ enhances the therapeutic efficacy of doxorubicin *in vivo* mouse model

Since our data suggest that suppression of autophagy by Rg_3_ can sensitize to doxorubicin-induced cell death, we carried out a subsequent experiment to test the effect of Rg_3_ on the therapeutic efficacy of doxorubicin *in vivo* using a mouse xenograft model. Huh-7 hepatocellular carcinoma cell xenografts were grown in athymic BALB/c nude mice As shown in Figure 6B, Rg_3_ or doxorubicin treatment alone had minimal effect on the growth of tumors at the doses used, with the tumor size similar to the control mice. Notably, combined treatment with Rg_3_ and doxorubicin led to significant reduction of the tumor volume (p value < 0.05 for combination treatment versus control, < 0.1 for combination treatment versus doxorubicin alone). Furthermore, no significant weight loss was observed during these periods of combination treatments (Figure S10). Consistent with the data on tumor volume, the tumor weight was also significantly decreased in combination of Rg_3_ and doxorubicin (Figure 6C). H&E staining of sections of the tumors from mice treated with the Rg_3_-doxorubicin combination showed that the cell density was greatly lower than in the Rg_3_- or doxorubicin- only groups (Figure 6D, top). Staining of tumor sections with Ki-67 and VEGF antibody revealed a much less intense staining in the mice that were given the Rg_3_-doxorubicin combination than in the other mice, indicating a reduction of cell proliferation and less angiogenesis in the tumor tissue from these mice (Figure 6D). TUNEL staining was highly evident in the Rg_3_-doxorubicin combination when compared to the Rg_3_- or doxorubicin- only groups. Taken together, these findings suggest that Rg_3_ potently enhances the therapeutic activity of doxorubicin *in vivo* by promoting cell death.

## DISCUSSION

It has been well established that autophagy possesses important biological functions and is closely implicated in oncogenesis and cancer development, where it can act in both tumor suppressive and pro-metastatic functions [57-60]. Suppression of autophagy has been accepted as a novel therapeutic strategy in combination cancer therapy [61], and may be especially useful in the treatment of HCC [14, 15]. Currently, chloroquine and its derivative, hydroxychloroquine (HCQ), are the only FDA approved drugs that are currently used in a clinical setting for inhibition of autophagy. Therefore, novel autophagy inhibitors for such combinational therapy in cancer are currently highly sought after.

20(S)-ginsenosides, a class of steroid glycosides and triterpene saponins, have been extensively studied as the main active components of ginseng [62]. Anti-tumor activity of the 20(S)-ginsenoside Rg_3_ has previously been associated with reduction of angiogenesis associated with down-regulation of VEGF expression [23, 24, 26-28]. We did not observe much VEGF downregulation in tumors from mice treated with Rg_3_ on its own, suggesting a different mechanism of anti-tumor activity in this case. Ginsenosides are able to affect multiple signaling pathways, and their effects on autophagy have remained uncharacterized. In the present study, we provide evidence that Rg_3_ has an inhibitory effect on autophagy that is functionally related to its sensitization effect on doxorubicin-induced cell death. Rg_3_ suppresses autophagy at a late stage via inhibition of the maturation, fusion, or degradation stage. Although the underlying molecular mechanisms for Rg_3_'s effects on autophagic flux remain to be further investigated, the effect of Rg_3_ on autophagy may be similar to that of CQ, which inhibits autophagy late in the process by interfering with lysosomal function. However, unlike CQ, the effect of Rg_3_ on autophagy may require changes in gene expression, and this may be mediated in part though activation of the CHOP transcription factor. CQ and HCQ are currently being investigated as primary or adjuvant therapies in more than 30 clinical trials for the treatment of cancer, mostly based on the premise that their anticancer properties are the result of inhibition of autophagy (see http://www.cancer.gov/clinicaltrials/search/results?protocolsearchid=9855259). HCQ and CQ have achieved favorable, but inconsistent, results in inhibiting autophagy in patients so far in phase I clinical trials [63]. The lack of CQ effectiveness in some trials may not be because inhibiting autophagy is not an effective strategy, but because CQ can be neutralized at acidic pH, eliminating its effectiveness in the hypoxic/acidic regions of tumors [64]. Therefore other compounds may need to be developed for effective inhibition of autophagy, and Rg_3_ may have the potential to be developed into a novel cancer therapeutic agent based on its ability to inhibit autophagy.

In this study we show that doxorubicin, a common cancer therapeutic agent that induces DNA damage [41], also induces autophagy, which plays a pro-survival function. Our findings are consistent with several other reports of doxorubicin-induced autophagy during cell death induction. For instance, low doses of doxorubicin have been recently shown to induce autophagy and apoptosis simultaneously [51]. In multiple myeloma cell lines, pharmacologic or genetic inhibition by knockdown of Beclin-1 or Atg5, augments doxorubicin-induced cell death [65, 66]. Our results show that inhibition of autophagy by Rg_3_ markedly augments doxorubicin-induced cell death in hepatocellular carcinoma cells *in vitro* and *in vivo*. Therefore, suppression of autophagy could be an effective novel strategy in combination therapy to overcome chemoresistance and enhance chemotherapeutic efficacy. In conclusion, Rg_3_ could potentially be further developed as an important autophagy inhibitor to enhance anti-cancer efficacy of existing DNA-damaging or other chemotherapeutic agents.

## METHODS

### Reagents

Anti-p62, anti-caspase-3, anti-Beclin-1, anti-Vps34, and anti-Atg5 antibodies were purchased from Cell signaling. Anti-LC3 antibody and necrostatin-1 were purchased from Sigma. Doxorubicin, chloroquine diphosphate (CQ), doxycycline, and cycloheximide were purchased from Calbiochem. zVAD was purchased from R&D system. 20(S)-ginsenoside Rg_3_ was isolated by prep-LC. Glutathione S-transferase (GST)-TRAIL was obtained as described previously [67].

### Cell culture

SK-Hep1, HepG2, A549, and H322 cells were cultured in DMEM supplemented with 10% fetal bovine serum, 2 mM glutamine, 100 U/mL penicillin and 100 μg/mL streptomycin. Huh-7 and Hep3B cells were cultured in RPMI 1640 medium supplemented with 10% fetal bovine serum, 2 mM glutamine, 100 U/mL penicillin and 100 μg/mL streptomycin. Atg5−/− MEFs and the Tet-off Atg5 MEFs were provided by Dr. N Mizushima (Tokyo Medical and Dental University). The normal liver cell line HL-7702 was purchased from Shanghai Institute of Cell Biology (Shanghai, China) and cells were cultured in RPMI 1640 medium supplemented with 20% fetal bovine serum, 2 mM glutamine, 100 U/mL penicillin and 100 μg/mL streptomycin.

### Western blot analysis

Upon treatment, cells were lysed in M2 buffer (20 mM Tris at pH 7, 0.5% NP-40, 250 mM NaCl, 3 mM EDTA, 3 mM EGTA, 2 mM DTT, 0.5 mM PMSF, 20 mM β-glycerol phosphate, 1 mM sodium vanadate, 1 μg/mL leupeptin). Equal amounts of cell extracts were resolved by 12% or 15% SDS-PAGE and analyzed by western blot and visualized by enhanced chemiluminescence (ECL, Amersham).

### Transfection

Transfection experiments in hepatoma cells were performed with Lipofectamine PLUS (Invitrogen, 11514) reagent following the manufacturer's instructions (GIBCO/BRL). Cells were transfected with the GFP-LC3 construct. The mRFP-GFP tandem fluorescent-tagged LC3 construct (tfLC3) was provided by Dr. T Yoshimori (Osaka University) [38].

### Confocal microscopy

Cells were seeded to a coverglass slide chamber (Lab-Tek®, NUNC), after designated treatments, the cells were examined under a confocal microscope (Carl Zeiss LSM710). GFP-LC3 puncta were examined by confocal microscopy and tfLC3 was also observed for change of both green and red fluorescence using a confocal microscope. The data presented were from one representative experiment of at least 3 independent repeats.

### Flow cytometry autophagy assay

For flow cytometric analysis, all cells were infected with pBABE retroviruses encoding mCherry-GFP-LC3 and selected for either puromycin or hygromycin resistance. Flow cytometry was done using 488 and 561 nM lasers for red and green fluorophore excitation, respectively. Fluorescent cell populations were originally enriched after selection by sorting the cells for red and green double positive cells on a Moflo XDP 100 machine (Beckman Coulter). Analysis was done on a Gallios 561 machine (Beckman Coulter). Any non-viable cells were excluded from analysis by gating on the appropriate forward/side scatter profile. The mCherry/GFP signal ratio of fluorescent cells was determined and then plotted as a histogram by WinList (Verity Software House). Since GFP is rapidly quenched by the low pH of the lysosome when autophagosomes merge with them, while mCherry is more stable to pH fluctuations, cells with high autophagy flux will be less green and thus have a higher mCherry/GFP ratio than cells with lower autophagic flux. Overnight treatment of cells with chloroquine or Bafilomycin A1 was done as a control to show that the mCherry/GFP fluorescence ratio properly decreased in the absence of autophagic flux, while treatment with EBSS was done to show that the ratio properly increased.

### Cytotoxicity assay

Cell death was determined using tetrazolium dye colorimetric test (MTT test). The MTT absorbance was then read at 570 nm. Lactate dehydrogenase (LDH) leakage was quantified using a cytotoxicity detection kit (Promega). The LDH absorbance was then read at 490nm. Representative images were taken by a phase-contrast microscope and the data presented are from a minimum of 3 independent experiments.

### Lentiviral shRNA experiments

MISSION short-hairpin RNA (shRNA) plasmids encoding sequences targeting Beclin-1 mRNA (NM-003766), ATG5 mRNA (NM-004849), Vps34 mRNA (NM-002647), and non-targeting control sequences (NC: SHC002) were purchased from Sigma-Aldrich. Lentiviral plasmids were transfected to 293TN cells (System Biosciences, LV900A-1) using Lipofectamine 2000 (Invitrogen, 11668019). Pseudoviral particles were collected 2 days after the transfection of lentiviral plasmids, and infected into SK-Hep1 and HepG2 cells in the presence of polybrene (8 μg/mL). Infected SK-Hep1 and HepG2 cells were selected for 2 days with puromycin (2 μg/mL), and knock-down confirmed by immunoblotting. Beclin-1 knock-down cells were re-infected with ATG5 or Vps34 pseudoviral particles respectively in the presence of polybrene. Double knock-down of Beclin-1/ATG5 or Beclin-1/Vps34 protein was confirmed by immunoblotting. Double knock-down cells were replated on 12 well plates and treated with doxorubicin for indicated time points and cells cytotoxicity was measured by MTT analysis.

### Tumor xenograft study

** Male nude mice from Central Lab. Animal Inc. (Seoul, Korea) were fed standard rat chow and tap water ad libitum, and were maintained under 12 h dark/light cycle at 21 ºC. Male, 6-week-old nude mice were randomly divided into four groups (control, Rg_3_, doxorubicin, Rg_3_+doxorubicin, n=8/group). Huh-7 cells were harvested and mixed with PBS (200 μL/mouse) and then inoculated into one flank of each nude mouse (5 x 10^6^ Huh-7 cells). When the tumors had reached a volume of about 50-70 mm^3^, mice were given a daily oral dose of 20 mg/kg Rg_3_ or the vehicle (200 μL PBS, control group), and i.p. three times/week at dose of 1 mg/kg doxorubicin, for 21 days, respectively. The tumor dimensions were measured twice a week using a digital caliper and the tumor volume was calculated using the formula: V = length x width^2^ x 0.5. Following the experiment, the mice were killed and the tumors were excised and weighed. Histopathological analysis for tumors was carried out by using hematoxylin and eosin (H&E) staining.

## SUPPLEMENTARY FIGURES


