# Supplementary material for: 20(S)-Ginsenoside Rg3 is a novel inhibitor of autophagy and sensitizes hepatocellular carcinoma to doxorubicin
Source: Oncotarget. 2014 May 28;5(12):4438–51. doi: 10.18632/oncotarget.2034 (PMC4147336; doi:10.18632/oncotarget.2034)
Supplement: Supplementary file 1 [file oncotarget-05-4438-s001.pdf]

20(S)-Ginsenoside Rg_3 is a Novel Inhibitor of Autophagy and Sensitizes Hepatocellular Carcinoma to Doxorubicin

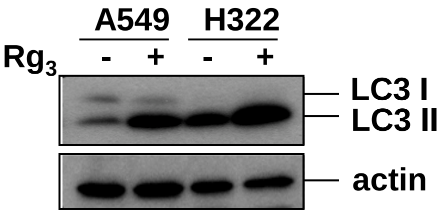

Supplemental Figure S1: Rg_3-induced LC3 II accumulation in lung cancer cell lines. A549 and H322 cells were treated with 100 µM Rg_3 for 12 h and cell lysates were analyzed by western blot.

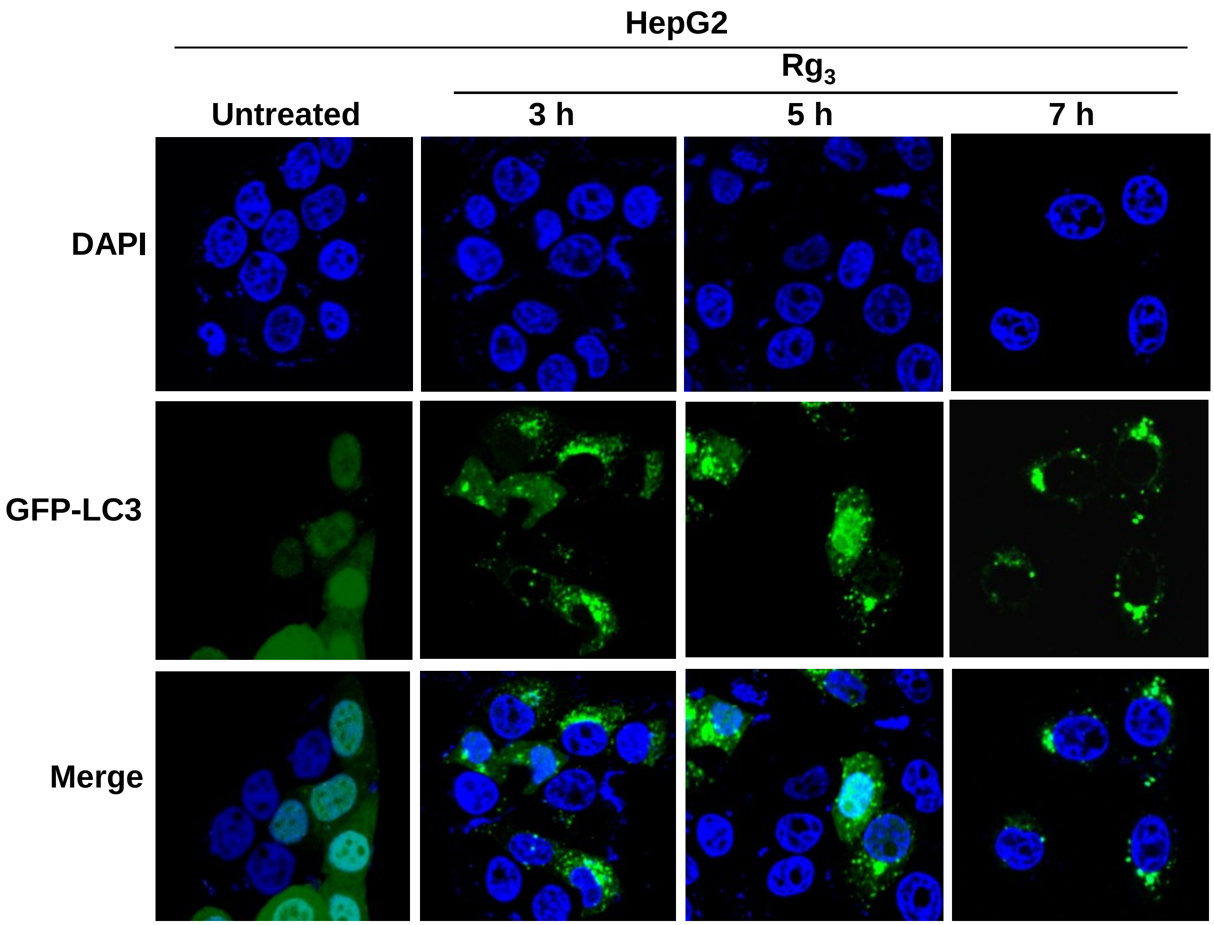

Supplemental Figure S2: Rg_3 treatment markedly increases GFP-LC3 puncta formation. HepG2 cells with transient expression of GFP-LC3 were treated with 100 µM Rg_3 for the indicated time pints and cells were stained with DAPI. GFP-LC3 punctuation /aggregation was observed under a confocal microscope.

A

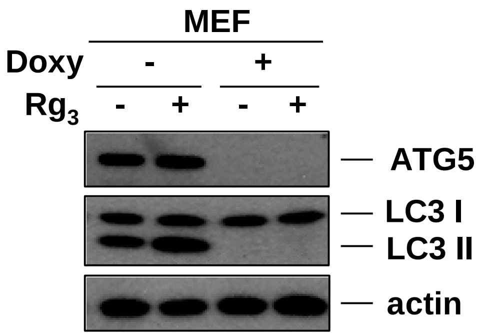

B

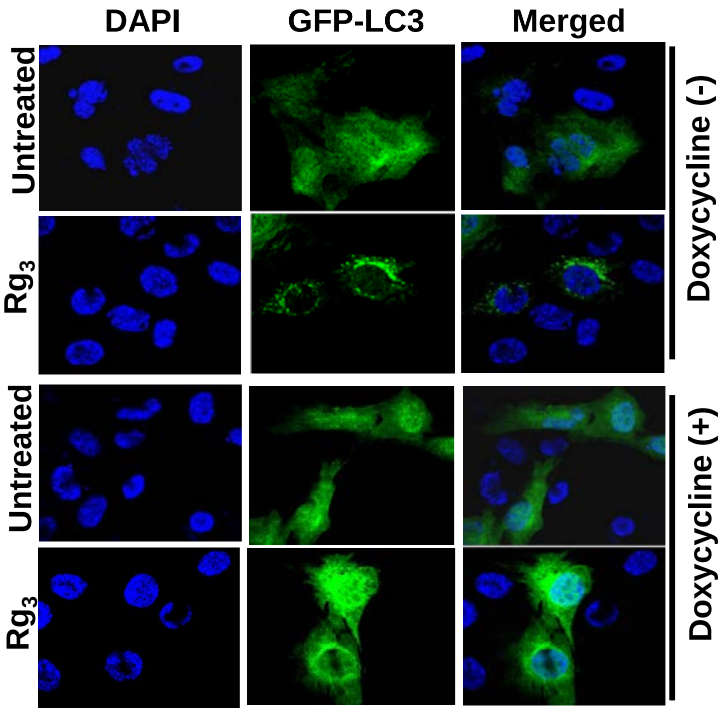

Supplemental Figure S3: A Rg_3-induced LC3 II accumulation is ATG5-dependent.

A. Tet-off Atg5 MEFs with stable expression of GFP-LC3 were pretreated with or without doxycycline for 4 days, then cells were treated with 100 µM Rg_3 for 12 h, and cell lysate was subjected to western blotting.

B. Tet-off Atg5 MEFs were prepared as described in (A), and treated with 100 µM Rg_3 for 3 h. Cells were examined with a confocal microscope for GFP-LC3 punctuation/aggregation.

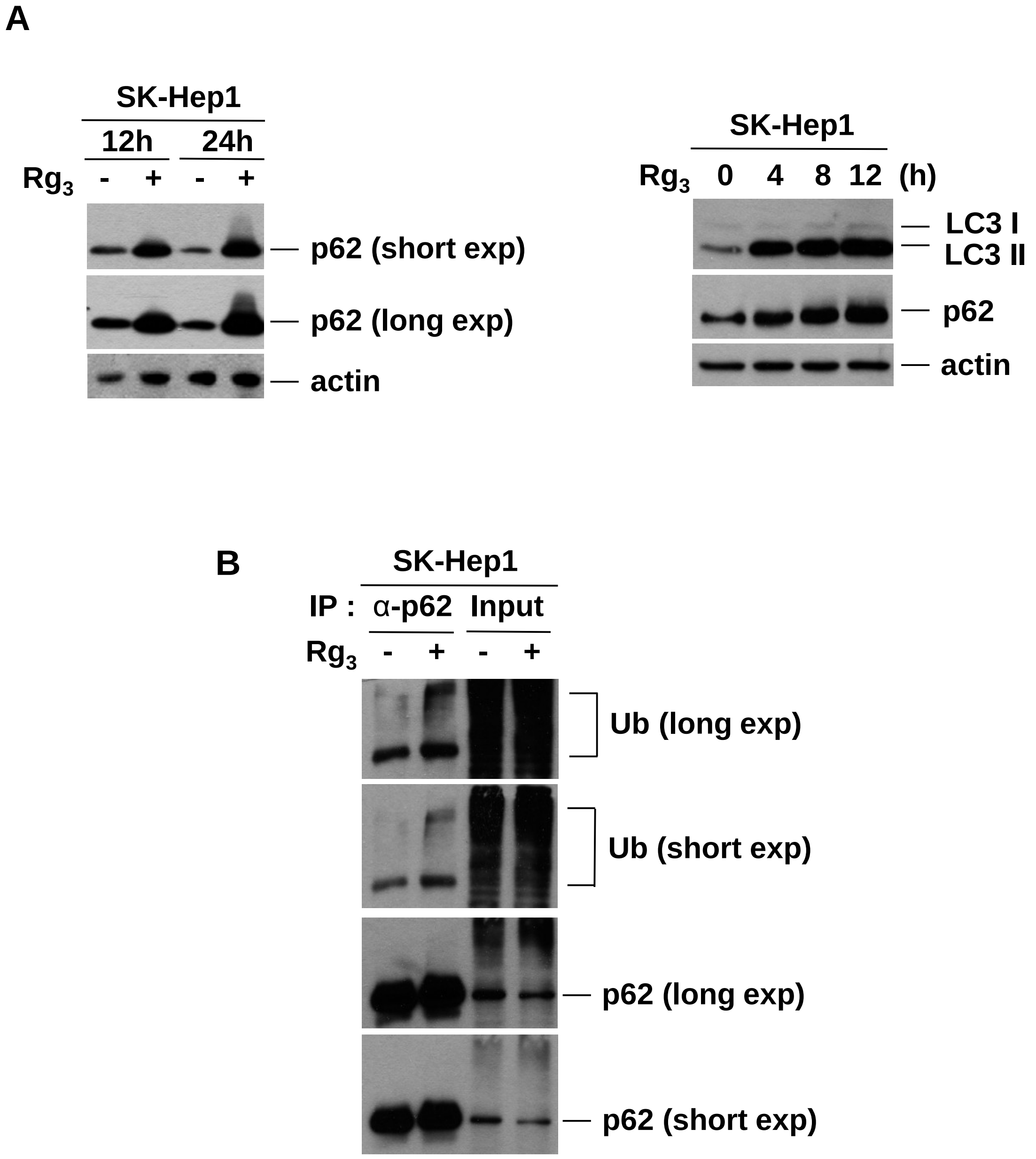

Supplemental Figure S4: Rg_3-inhibits p62 degradation in HCC cell lines.
SK-Hep1 cells were treated with 100 µM Rg_3 for the indicated times (A) or overnight (B) and cell lysates were analyzed by western blot (A) or immunoprecipitated with an anti- p62 antibody (B) and then analyzed by western blot with a ubiquitin antibody. Smearing in the Rg_3-treated lanes (A) suggests accumulation of ubiquitinated p62, which is confirmed by the ubiquitin western upon immunoprecipitation of p62 (B).

A

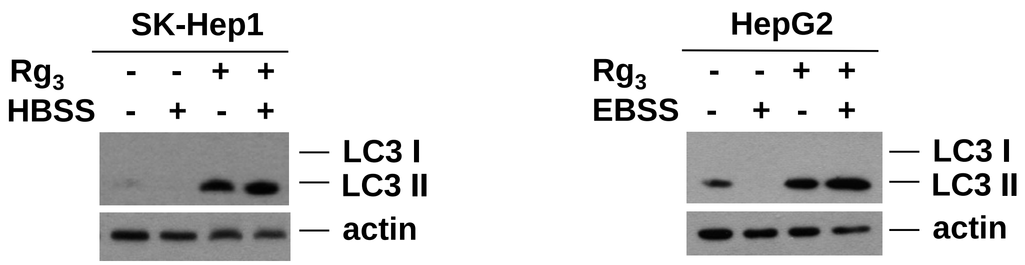

B

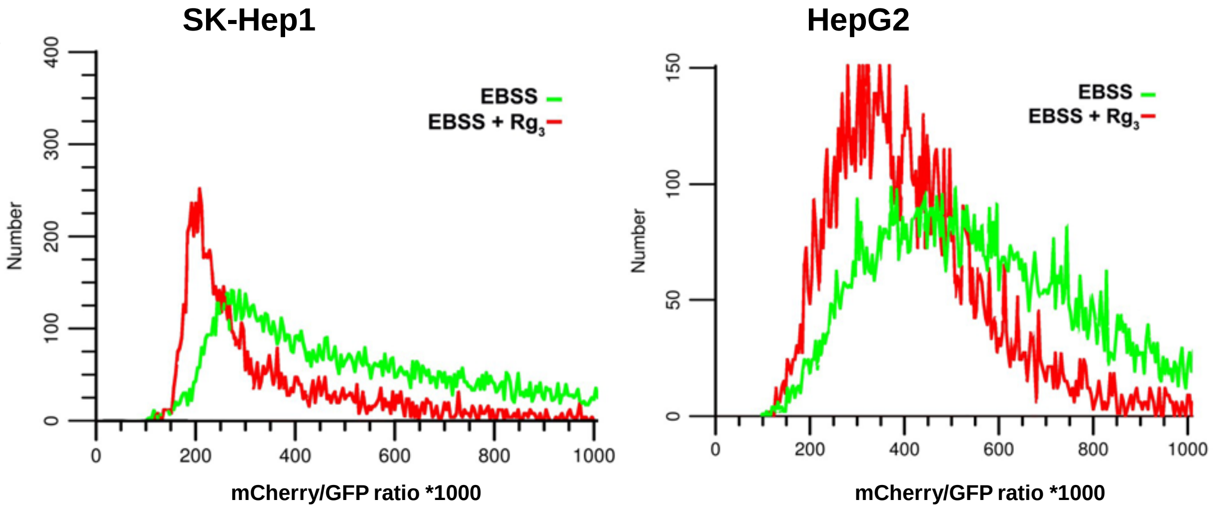

Supplemental Figure S5: Rg_3 inhibits starvation induced autophagy in HCC cell lines. (A) SK-Hep1 cells or HepG2 cells were pretreated with 100 µM Rg_3 for 1h and then treated with HBSS or EBSS for 4 hours and lysates analyzed by western blotting. (B) SK-Hep1 cells or HepG2 cells stably expressing tandem mCherry-EGFP-LC3 were pretreated with 100 µM Rg_3 for 1h and then treated overnight with EBSS and the Red/Green fluorescence ratio of the cells was analyzed by flow cytometry.

A

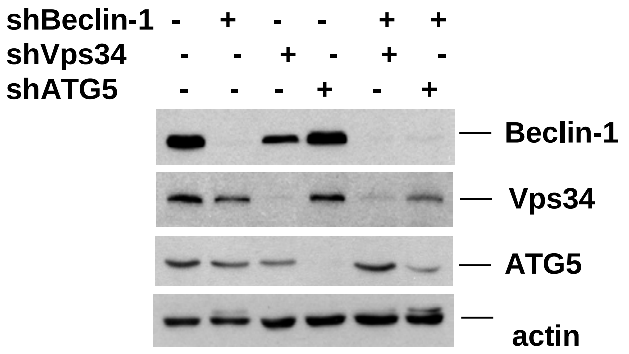

B

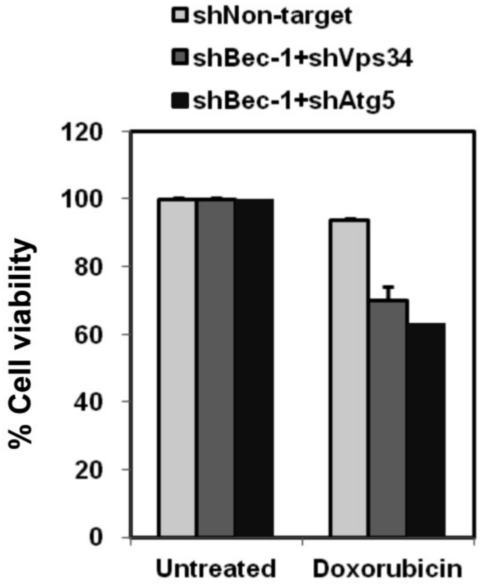

Supplemental Figure S6: Autophagy has a pro-survival role in doxorubicin-induced cell death.

A. Knock-down of autophagy-related genes. HepG2 cells were infected with a combination of lentivirus encoding Beclin-1, ATG5, or Vps34 shRNAs, or combinations thereof. After selection with puromycin (2 µg/mL), lysates were examined for knockdown by western blotting.

B. Suppression of autophagy by knock-down of Beclin-1/Atg5 or Beclin-1/Vps34 significantly enhances doxorubicin-induced cell death. Cells from (A) were treated with doxorubicin (2.5 µM) for 16 h and cell viability was analyzed by MTT assay. Results are averages +/- SEM.

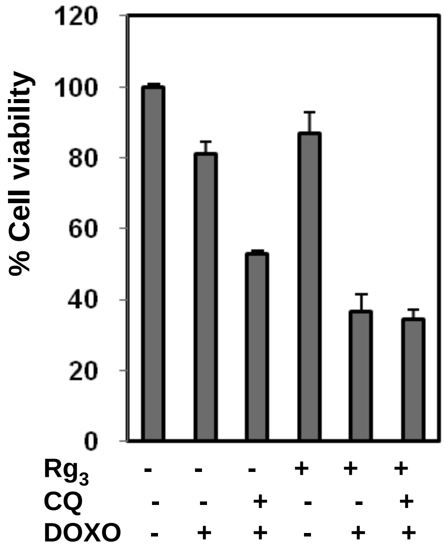

Supplemental Figure S7: Rg_3 can sensitize to Doxorubicin-induced cell death in a manner similar to chloroquine. The viability (MTT assay) of HepG2 cells was measured when pretreated with CQ (10 µM) or/and Rg_3 (100 µM) for 30 min, followed by doxorubicin (2.5 µM) for 18 h. Results are averages +/- SEM.

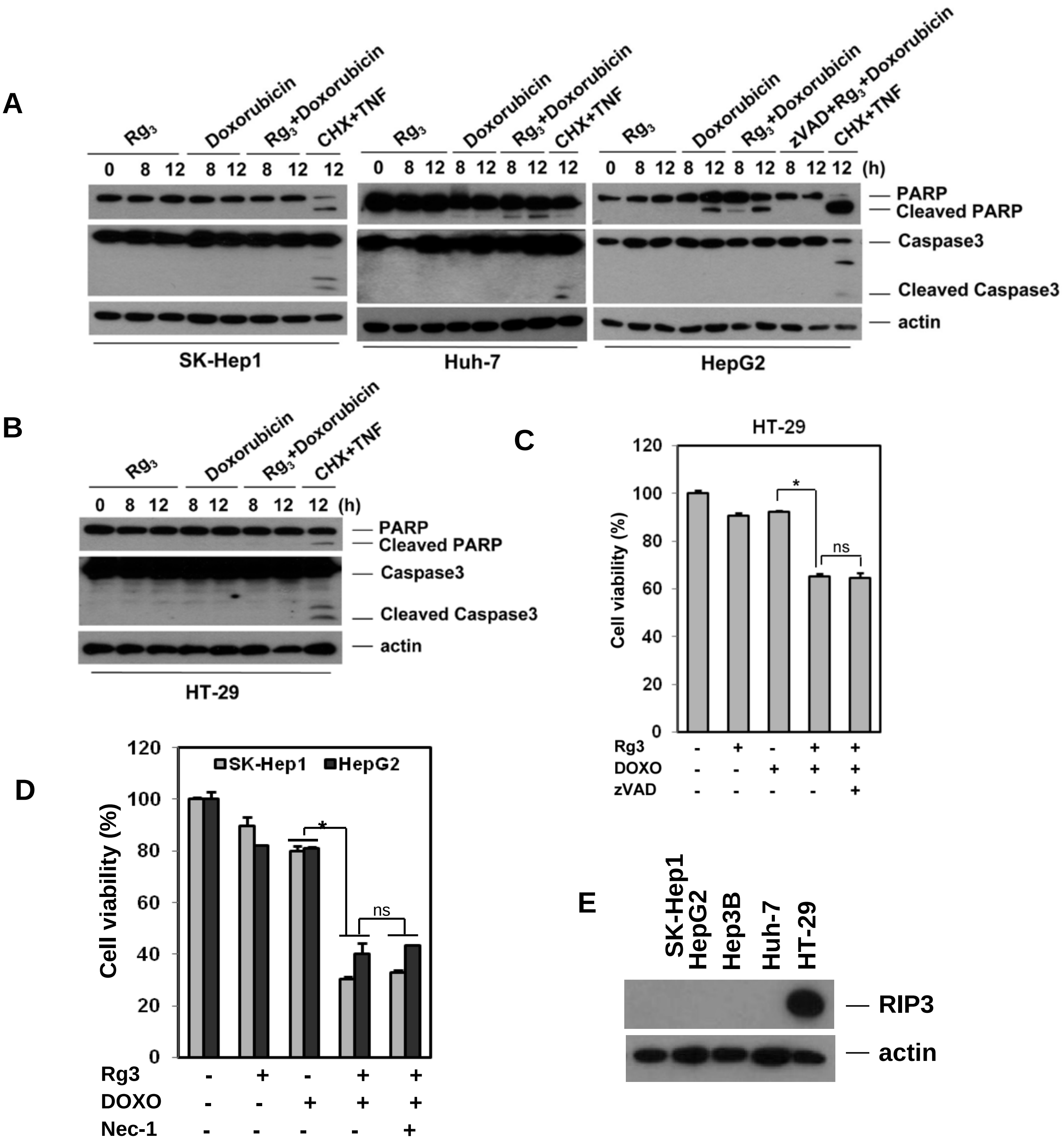

Supplemental Figure S8: Rg_3 and doxorubicin induced-cell death is caspase-independent in HCC cell lines. (A,B) SK-Hep1, Huh-7 or HepG2 cells (A), or HT-29 cells (B), were treated with Rg_3 (100 µM), doxorubicin (2.5 µM), or Rg_3 + doxorubicin for indicated time periods and or were treated with GST-TRAIL (25 ng/mL) for 12 h and cell lysates were subjected to western blotting for PARP cleavage and cleavage of caspase-3. (C) HT-29 cells were treated with Rg_3 (100 µM), doxorubicin (2.5 µM), or the combination Rg_3 + doxorubicin with or without zVAD (20 µM) for 18 h and cell viability was an analyzed by MTT assay. Results are averages +/- SEM. * p<0.001. (D) SK-Hep1 or HepG2 cells were treated with Rg_3 (100 µM), doxorubicin (2.5 µM), or the combination Rg_3 + doxorubicin with or without necrostatin (40 µM) for 18 h and cell viability was an analyzed by MTT assay. Results are averages +/- SEM. * p<0.001. (E) HCC cell lysates were probed by western blotting for RIP3 expression.

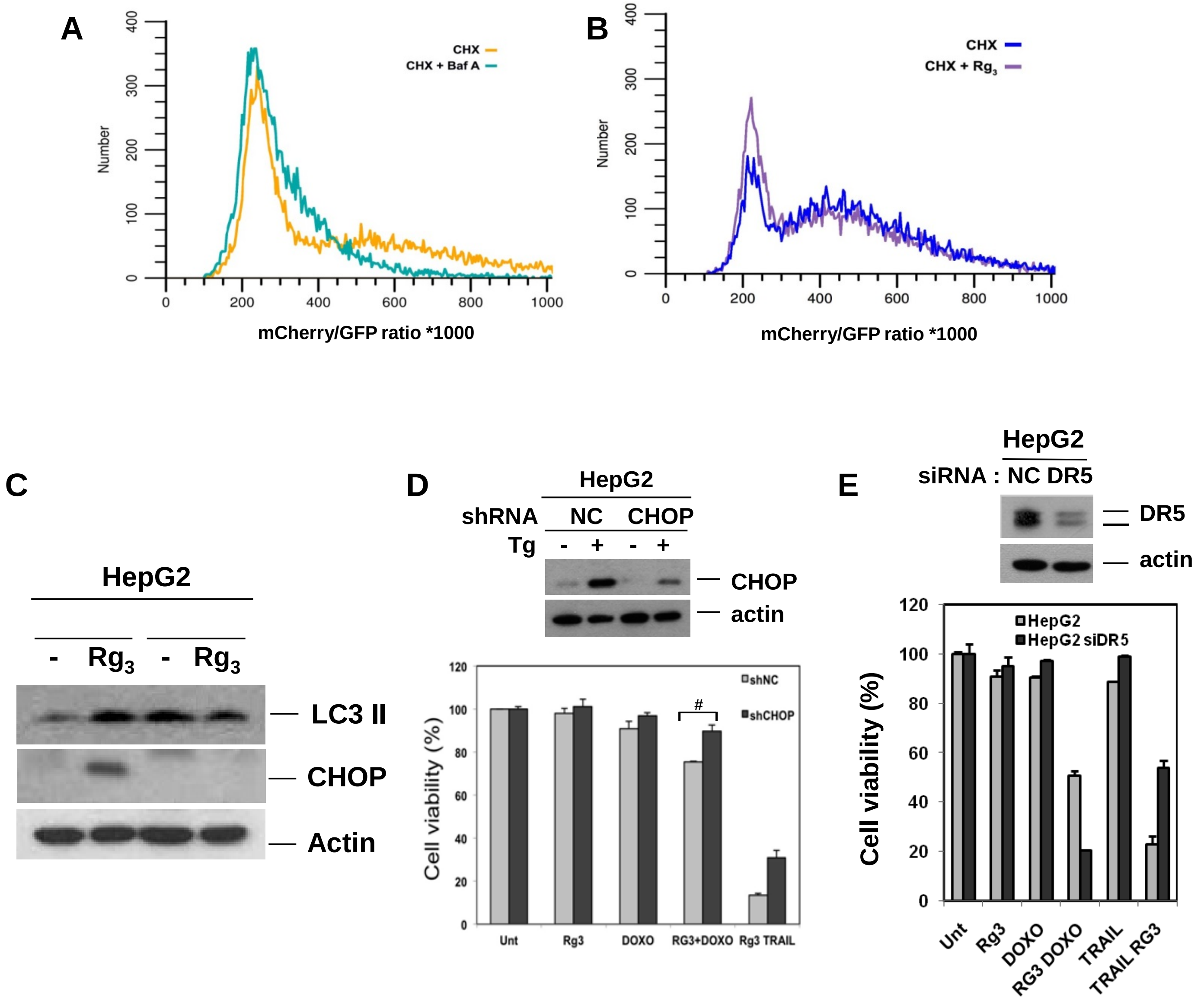

Supplemental Figure S9: Rg$_3$ suppression of autophagy may require induction of CHOP. (A,B) SK-Hep1 cells stably expressing tandem mCherry-EGFP-LC3 were pretreated with 5 µM cycloheximide for 1h and then some cells were treated overnight with (A) 10nM BafA or (B) Rg$_3$ (100 µM) and the red/green fluorescence ratio of the cells was analyzed by flow cytometry. (C) HepG2 cells expressing a CHOP shRNA hairpin or nonsilencing control were treated with Rg$_3$ (100 µM) overnight and lysates were analyzed by western blotting. (D) HepG2 cells expressing a CHOP shRNA hairpin or nonsilencing control were treated with Rg$_3$ (100 µM), doxorubicin (2.5 µM), the combination Rg$_3$ + doxorubicin, or or GST-TRAIL (25 ng/mL) for 12 h and analyzed by MTT assay. The Results are averages +/- SEM. $^{\#}$ p= 0.013. To show knockdown efficiency, some CHOP knockdown cells were treated with thapsigargin (1 µg/mL) for 12 h to induce CHOP expression. (E) HepG2 transfected with DR5 siRNA(48 hrs) were treated with Rg$_3$ (100 µM), doxorubicin (2.5 µM), or the combination Rg$_3$ + doxorubicin or TRAIL (25 ng/mL), or Rg$_3$ plus TRAIL for 16~18 h and viability was analyzed by MTT assay.

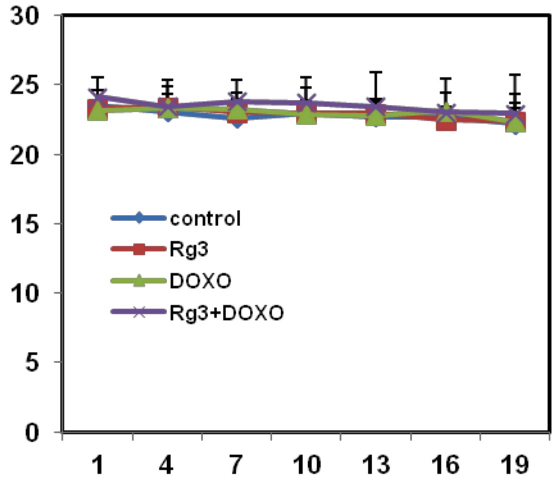

Supplemental Figure S10: Effect of Rg_3 plus doxorubicin on Huh-7 hepatocellular carcinoma cell xenograft in athymic BALB/c nude mice. Huh-7 tumors were established subcutaneously and treated with Rg_3, doxorubicin or Rg_3 plus doxorubicin for 19 days. Body weight was monitored.
